# *Mycobacterium* abscessus sporotrichoid infection after a dog bite^⋆^

**DOI:** 10.1016/j.abd.2023.02.004

**Published:** 2023-09-27

**Authors:** Patricia Guadalupe Mendoza-Del Toro (Patricia), Arturo Robles-Tenorio (Arturo), Víctor Manuel Tarango-Martínez (Victor)

**Affiliations:** a Department of Dermatology, Dermatological Institute of Jalisco “Dr. José Barba Rubio”, Secretariat of Salud Jalisco, Zapopan, Jalisco, Mexico; b Department of Dermatology, Faculty of Medicine and Health Sciences, Tecnologico de Monterrey, Monterrey, Mexico

Dear Editor,

The terms “atypical mycobacteria” or “non-tuberculous mycobacteria” (NTM) refer to a group of mycobacteria other than *Mycobacterium tuberculosis and Mycobacterium leprae*.^1^, ^2^ NTM are ubiquitous organisms that can resist extreme temperature conditions.^1^, ^2^ *M. abscessus* is a fast-growing atypical mycobacterium that can cause cutaneous lesions and disseminated infections, typically after skin trauma.^2^ Dermatological manifestations include nodules, abscesses, and ulcers that may often resemble *Sporothrix* infections.^1^ Zoonotic transmission is rarely reported.^3^

## Case report

**Figure 1:**
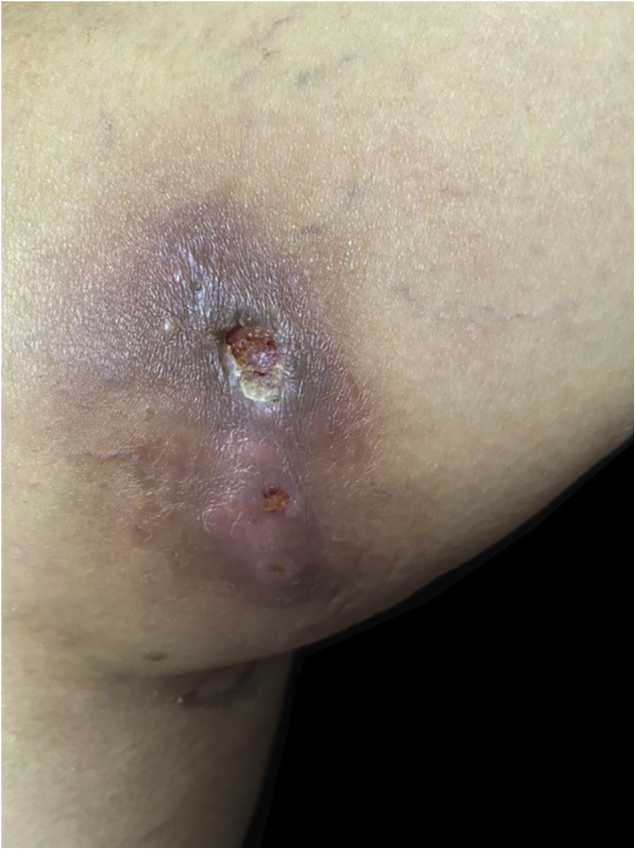
Three ulcerated, purulent, gummas affecting the thigh

**Figure 2:**
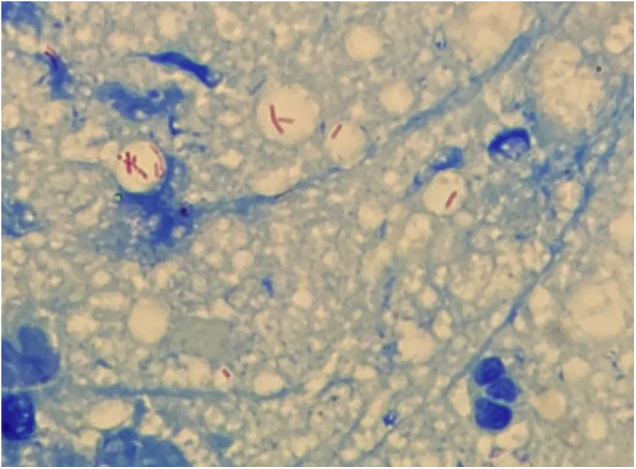
Abundant acid-fast bacili can be observed with Ziehl-Neelsen stain

**Figure 3:**
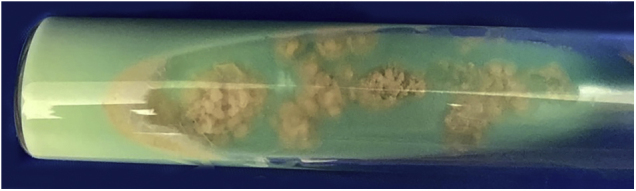
*M. abscessus* colonies grew in Lowenstein Jensen culture after 7 days

**Figure 4:**
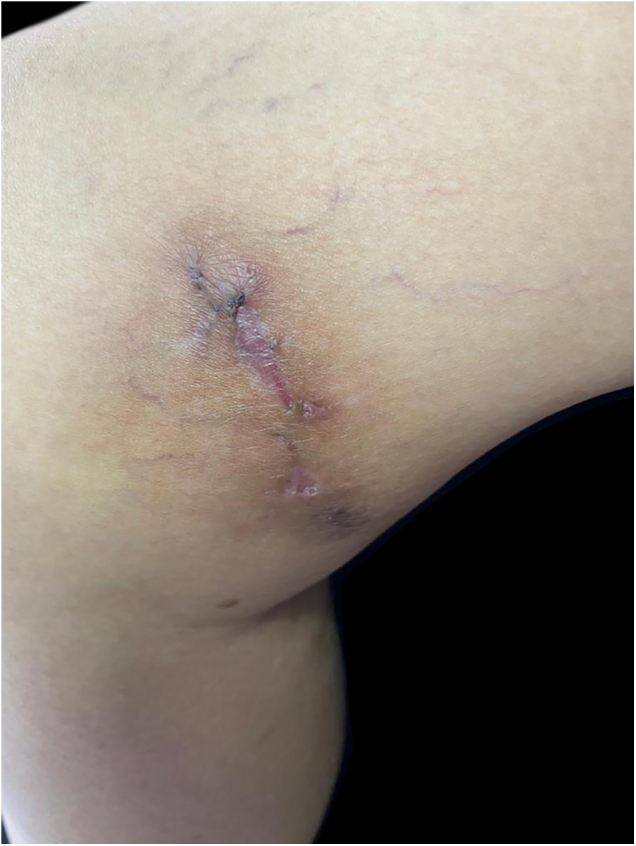
Favorable clinical response

A 63-year-old female presented with a history of a 2-month enlarging nodule over the medial aspect of the thigh after suffering a street dog bite. She was previously treated with clindamycin 300 mg TID for 21 days without improvement. On examination, there were 3 ulcerated, purulent gummas over an erythematous, warm, tender, fluctuating area of the thigh (Fig. 1). No adenomegalies were found. Samples were taken from the secretion for microbiological and molecular biology studies. Gram stain, KOH smear, and Sabouraud culture were negative. Acid-fast bacilli were identified on the Ziehl-Nielsen stain (Fig. 2). Creamy, white, cerebriform colonies grew on Lowenstein Jensen culture (Fig. 3) and with the identification of 1) PCR-RFLP (polymerase chain reaction-restriction fragment length polymorphism) of the gyrB and hsp65 genes with the digestion of the RsaI, TaqI or Sac II enzymes and HhaI enzyme respectively and 2) Multi-primer PCR to detect the absence or the presence of the RD9 and RD1 regions confirmed M. abscessus.The patient received amikacin 1 g IM daily for 2 weeks in two cycles plus clarithromycin 500 mg BID for 4 months, showing a favorable clinical response (Fig. 4).

NTM comprise a heterogenous group of acid-fast bacilli that are further classified according to their growth speed, morphology, and pigmentation.^1^ They were first described in 1931 by Pinners, and there are now more than 200 reported species.^3^ *M. abscessus* is associated with a wide spectrum of dermatological lesions that include cellulitis, abscesses, papules, pustules, fistulae, ulcers, necrotic lesions, and subcutaneous nodules that can resemble *Sporothrix* lesions.^2^, ^3^

Wounds by animal bites are typically caused by domestic dogs, which harbor a diverse oral microbiota that includes facultative and obligate anaerobes. Such diversity depends on the age, feeding, and oral health of the canine, among other factors.^4^ There are scant reports of zoonotic transmission of NTM infection after canine bites in both humans^5^, ^6^, ^7^ and dogs^8^, ^9^ (Table 1).^5^, ^6^, ^7^, ^8^, ^9^ In humans, only the upper extremity has been reported to be affected with either *M. fortuitum*, *M. kansasii*, or *M. chelonae*. In canines, only *M. fortuitum* and *M. smegmatis* infection have been documented. To the best of our knowledge, *M. abscessus* transmission has not been previously reported.

**Table 1:** Cases of non-tuberculous mycobacteria infection after dog bites in humans and canines

Author; year	Host	Comorbity	Affected area	Type of infection	Agent
Ariel et al; 1983.^5^	Male, 55 years old	None	Hand	Granulomatous synosivitis	*Mycobacterium fortuitum*
Southern; 2004^6^	Male, 68 years old	Diabetes mellitus type 2	Hand	Tenosinovitis	*Mycobacterium kansasii*
Minato et. al., 2021^7^	Male, 77 years old	None	Hand	Tenosinovitis	*Mycobacterium chelonae*
Fox et al., 1995^8^	Canine, male, 15 months old	None	Neck and trunk	Subcutaneous infection	*Mycobacterium fortuitum*
Malik et al., 2004^9^	Canine, female, 4 years old	Obese	Neck	Pyoderma panicullitis	*Mycobacterium smegmatis*
Malik et al. 2004^9^	Canine, female, 5 years old	Obese	Trunk	Pyoderma	*Mycobacterium smegmatis*

The diagnosis of NTM infection includes direct microscopy, culture in a selective medium, biochemical testing, chromatography, and molecular biology techniques; the latter being the preferred method.^10^

Unfortunately, the treatment is not well established. However, antibiotic combination therapy and surgical drainage of the lesions is strongly recommended.^1^, ^3^ The choice of antibiotics usually involves clarithromycin or azithromycin plus amikacin, cefoxitin, or imipenem for severe infections. Treatment duration may span from 3 to 6 months. There are no clinical studies that compare different treatment schemes.^2^

## Conclusion

Among all infectious agents that may cause disease after a canine bite, NTM is some of the most therapeutically challenging. The oral cavity of dogs may be colonized by NTM as a commensal pathogen and canines should therefore be considered a possible vector. Since the clinical presentation of sporotrichosis may be undistinguishable from NTM lesions, we emphasize the need to run all appropriate microbiological tests available that include acid-fast bacilli detection, apart from ordinary smears and cultures.

## Financial support

None declared.

## Authors' contributions

Patricia Guadalupe Mendoza-Del Toro: Critical literature review, preparation and writing of the manuscript.

Arturo Robles-Tenorio: Manuscript critical review, intellectual participation in propaedeutic and/or therapeutic management of studied cases.

Víctor Manuel Tarango-Martínez: Critical literature review, intellectual participation in propaedeutic and/or therapeutic management of studied cases, approval of the final version of the manuscript.

## Conflicts of interest

None declared.
